# Response to Drs. Heitzmann and Taft

**Published:** 1892-11

**Authors:** J. Leon Williams

**Affiliations:** 36 Bruton Street, Berkeley Square, London, W.


### Foreign Correspondence.

### RESPONSE TO DRS. HEITZMANN AND TAFT.

TO THE EDITOR OF THE INTERNATIONAL DENTAL JOURNAL:

SIR,—Will you allow me space for a few words with reference to two of the articles in the September number of your journal? It will be remembered that I read a paper before the First District Dental Society of New York in December, 1885, on "The Development and Minute Anatomy of the Teeth in Health and Disease." At the close of that paper I gave a summary of the arguments in the form of eight definite statements of facts, showing the fallacies of Heitzmann's theories of dentine and enamel formation, together with the views of retrograde metamorphosis, inflammation of dentine, "return to embryonal condition," etc., involved in those theories. Professors Heitzmann and Abbott were present on that occasion, and endeavored by resorting to specious arguments to draw attention from the force of the arguments and facts which I had presented. Not one of those set forth in this summary has ever been answered. But Professor Heitzmann and his pupils have from time to time serenely repeated these statements, disdaining all the facts against them, with their pet theory advanced from some slightly different point of view. The latest demonstration—the paper referred to above—comes from the pen of the great master himself, and in this article facts which are familiar to every one who has given any serious attention to the microscopic morphology of the teeth are advanced as something new and original, and confirmatory of the "reticular structure theory" of dentine and enamel. Professor Heitzmann says that Bödecker was the first to maintain that dentine has a reticular structure, and that "the positive proof that Bödecker's assumption was correct was furnished much later, in 1887, by William Carr." I imagine that Dr. Carr will not take the matter very much to heart on finding this honor stripped from him, but the fact is, that more than four years before this time I had exhibited, in Heitzmann's laboratory, sections of dentine treated with chloride of gold and acetic acid. Heitzmann was greatly delighted with these specimens, and pronounced them the finest illustrations of the "reticular" theory that he had ever seen. Now, in these specimens there was not a single novel feature. There was nothing that I had not seen hundreds of times before, and no appearance but such as every microscopist is perfectly familiar with. The sections had been cut from near the periphery of the dentine, where it joins the cementum, at which point there is often a fine branching of the dentinal fibrilla. I am not aware that any one has ever denied the existence of such branching or that it occasionally occurs in other parts of the dentine. The point to which I especially wish to call attention is, that no matter what appearance of branching or connection between the canaliculi they may or may not have seen, they have never shown anything *under the microscope or on paper* which can possibly be construed into a confirmation of their theories of the formation of dentine and enamel and the changes which occur in the decay of these tissues. At the same time I would call attention to the very marked difference in appearance of these last illustrations drawn by Professor Heitzmann, and those illustrating Dr. Bödecker's articles in the *Dental Cosmos* for 1878–79, a difference which the different methods of engraving totally fail to explain. As Dr. Allen very truly remarked, there is nothing shown except the ordinary branching of the canaliculi. I may further say that I can indorse all that Dr. Allen has said about the views of eminent scientific men abroad on this question. The recent important investigations of Mr. J. Howard Mummery, of London, serve to further confirm the fallacies of Heitzmann's teachings.

In Dr. Taft's paper on the use of "high potency" preparations of silica in the treatment of alveolar abscess there is much material for reflection. I long ago passed the age of absolute certainty, and, in the words of our dear old teacher, who has "passed beyond these voices," I no more "cry fool" at the dictum of any one; and yet, although I often learn from a child, I can no longer assent, with the happy, blind faith of childhood, to every proposition placed before me. I know people who seem to be sensible on most questions who profoundly believe that they have shaken hands with the spirit of their grandmother. On the other hand, a friend of mine is very sceptical on the subject of spiritualism, but has unbounded faith in the "Keely motor." Some very scholarly people are convinced that there is great efficacy in "Christian science" as a method of cure; and has not the good bishop of Treves just testified on oath that he witnessed many miracles last year at the exhibition of the "holy coat"? Alas that some of us should find these things an offence to "that noble and most sovereign reason," when there seems so much contentment in believing!

I, too, have had my experiences in homœopathy. I am sorry to say that I once devoted the best part of two years to study and experiment in this field. Decided effects seemed sometimes to result from the use of "high potency" preparations, and great was my faith in the ten-thousandth attenuation of calc. carb., until an unlucky day when I discovered that startling effects seemed sometimes to be produced when only *water* had been administered. But my complete abandonment of the use of high attenuations dates from the time when I discovered that I must either give up this method of treatment or avow my disbelief in the atomic and molecular theories of chemistry. One of the greatest physicists of modern times has given us the extremes between the limits of which we may find the measurement of a molecule of hydrogen.[1] Now, if we begin with a grain of silica, we shall find that long before we have reached the

---

[1] I am writing this from memory, and must be allowed some small latitude in form of expression.

hundred-thousandth attenuation we have parted with the last remaining molecule of the original substance. But to suppose that silica, or any other substance of like character, can be reduced to a molecular condition by trituration in a mortar, is, of course, too apparent an absurdity for even the most credulous. No substance can in any such way be reduced beyond the point of discovering its smallest particles with a microscope. Any one can, therefore, see on a moment's thought that even before the ten-thousandth decimal attenuation were reached, one portion of the liquid menstruum would hold the last particle, and the other portion would be quite innocent of the wonderful dynamic-power-substance. I wonder if any of the eminent gentlemen who discussed Dr. Taft's paper stopped to consider the meaning of the progressive division of a unit by ten until the hundred-thousandth division be reached? I write a rather fine hand, and I find that the nine ciphers required with the unit to express one billion measure about one inch, as I usually write them. According to this rate of measurement, the denominator of a fraction (having one for its numerator) written to express that part of the original grain of silica remaining when the hundred-thousandth decimal attenuation is reached, would extend something over *nine hundred feet.* In comparison with this, how insignificant seem the awful mathematical calculations with which lecturers on astronomy are fond of paralyzing the minds of their hearers! Dr. Andrews is reported as saying that "the action of homœopathic remedies does not seem at all improbable to those who have prepared microscopical tissues and have noted the effect that minute percentages of reagents have on them." I have had the pleasure of looking at many of Dr. Andrews's beautiful microscopic sections, but I imagine that if the staining had been done with a dye attenuated to the point of Dr. Taft's silica, they would command but little admiration as specimens of microscopical coloring. A drop of almost any staining agent known, mixed with all the water that passes over Niagara Falls in a day, would make a brilliant dye in comparison with the attenuation mentioned. Seriously, the field for legitimate scientific experiment in matters connected with dentistry is so broad, and there is such need for real scientific work, that it seems a pity to waste valuable time in discussing ridiculous impossibilities.

Fraternally yours,

J. Leon Williams.

36 Bruton Street, Berkeley Square, London, W.,
September 23, 1892.

